# The Next Generation of Interoperability Agents in Healthcare

**DOI:** 10.3390/ijerph110505349

**Published:** 2014-05-16

**Authors:** Luciana Cardoso, Fernando Marins, Filipe Portela, Manuel Santos, António Abelha, José Machado

**Affiliations:** 1 Computer Science and Technology Center, Department of Informatics, University of Minho, Campus de Gualtar, Braga 4710-057, Portugal; E-Mails: a55524@alunos.uminho.pt (L.C.); a55561@alunos.uminho.pt (F.M.); abelha@di.uminho.pt (A.A.); 2 Algoritmi Center, Department of Information Systems, University of Minho, Campus de Azurém 4800-058, Guimarães, Portugal; E-Mails: cfp@dsi.uminho.pt (F.P.); mfs@dsi.uminho.pt (M.S.)

**Keywords:** healthcare interoperability, Agency for Integration, Diffusion and Archive of Medical Information(AIDA), multi-agent systems, agent monitoring

## Abstract

Interoperability in health information systems is increasingly a requirement rather than an option. Standards and technologies, such as multi-agent systems, have proven to be powerful tools in interoperability issues. In the last few years, the authors have worked on developing the Agency for Integration, Diffusion and Archive of Medical Information (AIDA), which is an intelligent, agent-based platform to ensure interoperability in healthcare units. It is increasingly important to ensure the high availability and reliability of systems. The functions provided by the systems that treat interoperability cannot fail. This paper shows the importance of monitoring and controlling intelligent agents as a tool to anticipate problems in health information systems. The interaction between humans and agents through an interface that allows the user to create new agents easily and to monitor their activities in real time is also an important feature, as health systems evolve by adopting more features and solving new problems. A module was installed in Centro Hospitalar do Porto, increasing the functionality and the overall usability of AIDA.

## 1. Introduction

Nowadays, one of the greatest concerns of organizations is to introduce technology into their environments. Health organizations are not an exception: health information systems (HIS) have been introduced in order to improve the quality of healthcare delivery [3]. Despite the fact that HIS contribute to a significant increase in health service quality, these systems are developed in an isolated way by different entities, which make different assumptions. This independence complicates the interaction among the different systems existing in an environment, making it complex and heterogeneous [4]. To solve these problems and to improve their overall performance and usefulness, it is necessary to implement proper communication and cooperation among these systems.

To accomplish these purposes, it is fundamental to incorporate into these environments the concepts of integration and interoperation at different conceptual levels and with different objectives. Both are important for cooperation and information flow in healthcare organizations; however, they are based on different principles. While integration aims to obtain information from several systems in order to improve their capabilities, interoperation focuses its goal on a continuous communication and information exchange among cooperating systems [2].

The main goal of interoperability in healthcare is to connect applications and data, so that they can be shared throughout the environment and distributed by health professionals. In this way, the information is always available and accessible in order to make health professionals’ workflow [1] easier.

The Agency for Integration, Diffusion and Archive of Medical Information (AIDA) is an agent-based platform that guarantees interoperability in several Portuguese hospitals [5]. However, it fails in controlling and monitoring its own agents. The module for controlling the agent community of AIDA as developed and presented in this paper arises precisely in order to overcome this flaw. Thus, the AIDA administrators can verify the agent functioning or detect possible failures in their operation in real time. This new module enables the AIDA administrators to perform better management of the whole platform. They can even decide the most appropriate period to make system routine changes, updates, maintenance and other operations, improving the overall performance of the AIDA. The AIDA platform with this new module will ensure interoperability and improve the quality of services in health institutions.

The methodology used in the development of this new AIDA module was design science research [6]. The first step of this methodology is to identify the problems and motivations—in this case, the difficulty in controlling AIDA agents and their activities. The second step of design science research is to delineate the objectives; in order to overcome the drawbacks of the AIDA mentioned above, the main features of a module for controlling the agent community of AIDA was studied (presented in Section 5). The design and development step is also presented in Section 5. The demonstration and evaluation steps are evidenced in Section 6, and the last step of the design research methodology (communication) is the revelation of this paper [6].

This paper is organized into seven sections. This first section gives a short introduction of the work, the subject in which it is embedded and the main objectives and motivations. Section 2 demonstrates important concepts for this work, specifically the importance of the interoperability in health organizations and the technologies and standards. Some agent-based solutions for interoperability are presented in Section 3 (Related Work). The AIDA platform is presented in Section 4, as well as a brief study of its advantages and disadvantages. Section 5 presents the module for controlling the agent community of AIDA in detail: its features, its architecture and the description of its components. The results obtained after the module for controlling the agent community of AIDA for implementation are presented and discussed in Section 6. Finally, Section 7 summarizes the main conclusions and presents some future work.

## 2. Interoperability in Healthcare

Interoperability is the ability of two parties, either human or machine, to access and to use the data reliably and quickly from various sources and systems in order to operate on them together without the occurrence of errors [7]. According to the Institute of Electrical and Electronics Engineers (IEEE), interoperability is “the ability of a system or a product to work with other systems or products without any additional effort on the part of the customer”. According to the International Organization for Standardization (ISO), another definition of interoperability is the ability of independent systems to exchange important information and to initiate actions on each other, aiming to work together for mutual benefit [2].

The interoperability among the HIS has a substantial role that enables these systems to communicate in order to share information, improving its high availability [2]. However, the interoperability implementation process in these systems is not a simple task. All transferred information must be normalized in order to avoid different structures and misunderstandings [8]. In this way, the use of standards ensures better communication between health professionals and interoperability among systems, allowing some automation of hospital records. These standards can be categorized depending on their purposes: standards for communication, standards to represent clinical information and image standards [9,10].

The standards in the health area are considered the main source for ensuring interoperability among the HIS [10]. The most used standard for communication is Health Level Seven (HL7) [11]. It is a set of formats that specify the interfaces for electronic data exchange between heterogeneous computer applications in hospital environments. The HL7 is centered in the syntax of the exchanged information. In addition, this standard defines that the information should be sent through a message, potentializing the use of HL7 in a client-server architecture. The actual version of HL7 (version 3), besides defining a syntax for the messages exchanged, also aims to achieve semantic interoperability, specifying the message content through an information model that clarifies the definitions, and it ensures that these definitions are used consistently [10,12].

Relative to the standards for representing clinical information, the terminology, Systematized Nomenclature of Medicine-Clinical Terminology (SNOMED-CT), stands out. The SNOMED-CT can be used by health professionals, administrators and researchers in the medicine area in order to improve the quality of healthcare delivery through an efficient and concise representation of clinical information. This terminology assists in the organization of medical terms, and it can be integrated with the electronic health record (EHR). In this way, the information is stored uniformly, aiding its processing and its automatic analysis [2].

The standard most used for medical images is Digital Imaging and Communications in Medicine (DICOM) [13]. This standard defines structures and data services for the exchange of medical images (from any modality) and related information. DICOM uses a binary encoding with hierarchical lists of data elements identified by numerical tags and a complex network protocol designed for the DICOM standard. In this way, the interoperability among different equipment is achieved, and the availability of the images and related information is guaranteed [12].

### 2.1. Principles of Interoperability

Besides the complexity of its implementation, there are also several principles associated with interoperability. There are several models that can be found that classify interoperation approaches based on a set of attributes, which define the exchanged data abstraction level, the interoperation viewpoint and the technological implementation [14]. Most authors consider only syntactic interoperability and semantic interoperability; however, in [7], human semantic and computable semantic is distinguished :

• Syntactic interoperability: allows the exchange of information among different systems or applications through a grammar. The entity that sends the information encodes it, respecting the syntactic rules of a specific grammar. On the other hand, the entity that receives the information decodes it using the same syntax rules.
• Human semantic interoperability: ensures that the meaning of the data is not ambiguous when exchanged between humans. In health environment, documents, such as progress notes, referrals, consultations and others, depend on the specificity of the clinician’s vocabulary, and it is common practice to ensure the semantic interoperability among clinicians.
• Computable semantic interoperability: enables the meaning of the data to not be ambiguous in exchanging data among the machines. The computable semantic does not require that all machines process the data received in the same way, but all of them have to interpret the meaning of the data equally.

The Dublin Core Metadata Initiative created an interoperability model constituted by four levels, and it is based on the data abstraction [14]:

• Level 1, shared terms definitions: definition of the sharing language of the data components.
• Level 2, formal semantics of interoperability: the data are based on the formal semantics.
• Level 3, set of descriptions of syntactic interoperability: the data are structured according to a formal vocabulary.
• Level 4, description of the profile interoperability sets: the data content is structured according to a formal vocabulary, and it is limited by a set of invariants.

**Figure 1:**
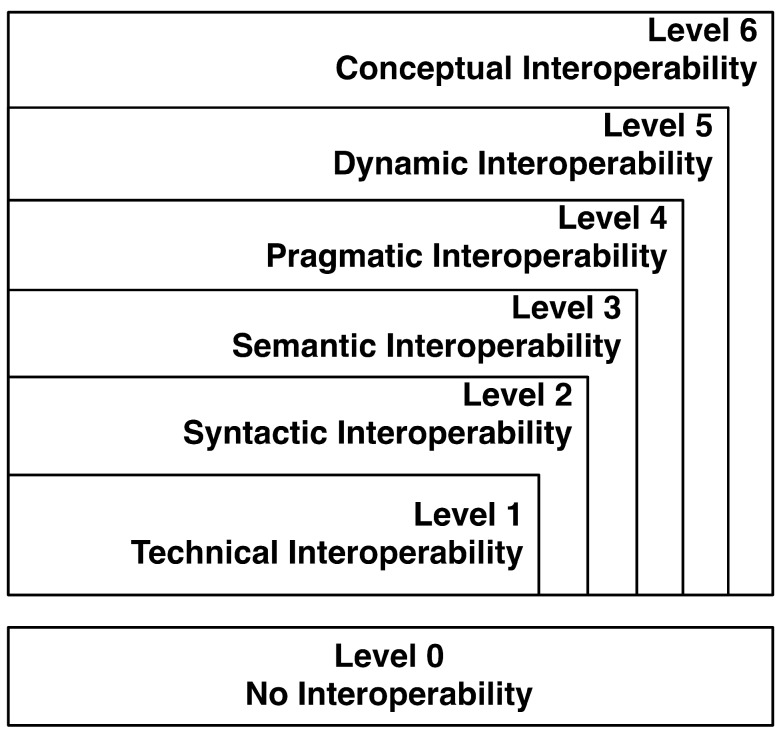
Levels of conceptual interoperability model (LCIM) adapted from [15].

Another model is presented by Tolk and Muguira [15], and it is not only based on the abstraction level of the data exchanged, but also uses methodologies (technology implementation) for problem solving, improving the interoperation process. This model is denominated by the levels of conceptual interoperability model (LCIM), and it has seven levels, wherein the lowest level (Level 0) represents the absence of interoperability and the highest (Level 6) a scenario with conceptual interoperability. These levels are presented in the Figure 1, and they are described in the next few lines [15]:

• Level 0: Stand-alone systems with no interoperability;
• Level 1: In technical interoperability exists a protocol for communication between systems, enabling the exchange of information through the network of well-defined protocols;
• Level 2: As already mentioned, when it reaches a level of syntactic interoperability, the systems have a common and well-defined structure regarding the format of the information that is exchanged;
• Level 3: Also already presented, the semantic interoperability is reached when the content of the information exchanged is interpreted in the same way on all systems;
• Level 4: In order to obtain pragmatic interoperability, it is necessary that the systems involved are aware of the methods and procedures that each system performs; the context in which the exchange of information is carried out is understood by all participating systems;
• Level 5: The states of the systems are modified as they operate on the data over time. If dynamic interoperability is achieved, the systems are able to perceive and to take advantage of state changes. The effects caused by the exchange of information are understood by all systems involved;
• Level 6: To achieve the highest level of the LCIM (conceptual interoperability), it is essential that the systems are in conformity with the assumptions and constraints of each real environment. To achieve this purpose, it is necessary to document the conceptual models through methods used in engineering, so that any engineer is able to understand them. When conceptual interoperability is achieved, the participating systems can be applied to different environments where the assumptions and constraints are different.

Still referring to Figure 1, it is important to note that as the level rises, the ability to interoperate among the systems is greater. As Figure 1 suggests, the Level 6 encompasses all the levels; Level 5 includes all levels below it, and so on. When conceptual interoperability is achieved, all the characteristics of the other levels are also incorporated into the system [15].

### 2.2. Multi-Agent Systems for Interoperability

There are various technologies capable of developing systems to ensure interoperability among HIS, for example Service-Oriented Architecture (SOA), Multi-Agent Systems (MAS), web service interfaces and Extensible Markup Language (XML) [2]. These technologies are often complementary instead of competitive. However, multi-agent technology has excelled in this topic [8,16]. This technology is closely related to the fundamental concepts that define a distributed architecture. Agent-based computing has emerged, due to its ability to solve problems and/or make a new revolution in the development and analysis of software [17].

It can be said that intelligent agents are understood as computational artifacts that exhibit certain properties, such as autonomy, pro-activity, reactivity and social skills [8]. They should be defined as troubleshooting entities of autonomous computing capable of operating effectively in a dynamic environment. They are often used in environments where they interact and cooperate in order to achieve a global goal. An intelligent agent is endowed with autonomous behavior, which allows it to decide and to execute its own actions, without direct human intervention [18]. The pro-activity allows one to plan and to perform tasks designed to achieve the proposed objectives. The social skills allow the interaction and cooperation of one agent with the others, which belong to the same multi-agent system. Finally, the reactivity enables the agent to be aware of the environment where it is inserted, and consequently, it reacts to stimuli captured by the sensors [17].

A MAS is a group of intelligent agents that inherits the properties described above. This technology is based on a distributed architecture, and it is essential that agents communicate with each other in order to ensure the interoperation among several applications, in this case, among the HIS. Through the lifecycle of an agent, the MAS can manage the availability of the modules integrated with the system and the HIS as a whole, keeping all the agents that compose the MAS freely distributed [18,19].

In this context, the reference model, Foundation for Intelligent Physical Agents (FIPA), has emerged as a standard for agent-oriented programming, which, among all its specifications, there is one that aims to standardize the communication among the agents in order to ensure their social skills and, consequently, their interoperability [20]. This specification, called Foundation for Intelligent Physical Agents-Agent Communication Language (FIPA-ACL) is a set of standards covering: the structure of an ACL message that can be used by agents to build their messages; a set of communicative acts (primitives) by which it specifies the types of ACL messages; and a set of communication protocols that support the interaction and exchange of messages [16,20].

In the development of the module presented in this paper, the MAS technology and the FIPA-ACL were used to ensure the uniformity of messages exchanged between agents.

## 3. Related Work

As referenced previously, multi-agent technology has been detached in the interoperability field, giving to information systems (IS) a greater reliability, flexibility, adaptability and maintainability [21,22]. For example, Marques [23] presents an interoperability architecture to be integrated with governmental services. This architecture is based on agents and web services, and its main goal is to provide a secure way to deliver integrated services to clients (citizens, businesses or public administration). Acampora [24] used an approach based on the integration of collaborative agents and mimetic algorithms to solve interoperability problems. Contreras presents Component Agent Platform based on NET (CAPNET) [25], an agent-based platform that enables the creation of a distributed multi-agent system with mobile agents, ensuring its communication and cooperation. Like this system, there are already several tools that provide an attractive environment to create multi-agents systems [26,27]. They are distinguished by the programming language and the standards they use.

In healthcare, agent-based systems are growing exponentially; however, there are some agent-based solutions for interoperability among HIS [21]. Lanzola [28] presents a methodology for the development of interoperable agents to be applied in medical applications. Tyson [29] presents an agent-based system for recruitment in clinical trials, and this author demonstrates that the agent-based approach has several potentialities over the client-server approach. Isern [22] proposes a general framework based on a MAS and knowledge representation technique to allow the enactment of clinical guidelines. Orgun [30] describes the implementation of an ontology and MAS-based system as a framework for the interactions among heterogeneous systems in a healthcare organization. Taweel [31] states that the implementation of semantic interoperability standards is not enough for complex data-intensive closed domains, such as the HIS. This author defends that an approach to dynamic semantic interoperability based on domain ontologies and extensible semantically-enriched problem models is a more effective solution, presenting a prototype. Kim [32] proposes a methodology for the design and implementation of convergence mobile agents to develop ubiquitous healthcare (u-healthcare) systems. Kaluža [33] reveals an MAS that aids elderly people, preventing falls and pointing out health problems through an intelligent environment enriched by sensors.

Several researchers in the area of hospital interoperability have used Java Agent Development Framework (JADE) technology, a library developed in Java that allows the implementation of MAS according to FIPA specifications to ensure the interoperability and scalability of heterogeneous environments, such as health institutions. Some of the systems with these characteristics have been shown in some studies [2,34,35]. For example, Astaraky [36] presents a decision support system based on an MAS to aid an interdisciplinary healthcare team in cases of patients with chronic illnesses. Other agent-based systems, also in the health field, have been developed. These tools are mostly clinical data management, decision support systems and systems that allow the remote monitoring of objects and patients [21].

## 4. Agency for Integration, Diffusion and Archive of Medical Information (AIDA) Platform

The Agency for Integration, Diffusion and Archive of Medical Information (AIDA) is a platform based on multi-agent technology that makes the HIS interoperable. AIDA was developed by a research group of artificial intelligence at the University of Minho and is already the main tool that ensures the interoperability in several Portuguese health organizations. This is the case of the Centro Hospitalar do Porto (CHP), the Centro Hospitalar do Tâmega e Sousa, the Centro Hospitalar do Alto Ave and the Unidade Local de Saúde do Norte Alentejano [5].

CHP is a public hospital. It is also a central health school, which aims at excellence in its activities, in a comprehensive and integrated health perspective. It focuses on the provision of care to improve the health of patients and the population in activities of high differentiation and support and the linkage with other health institutions. It also provides privileges to pre- and post-graduate education and encourages research with the aim of contributing to the development of science and health technology. The AIDA platform is based on the agent-oriented paradigm, and it has demonstrated great adaptability, modularity and effectiveness, once it becomes a basic multi-agent system that grows according to the particular needs of each institution [17]. This platform was designed to assist medical applications and to control the flow of information through a network of intelligent information processing systems with an adjustable level of autonomy. Its main objective is to allow the diffusion and integration of the information generated in a hospital environment [8]. In this way, the information that is stored from various sources is automatically available to authorized authorities, when and where they need it. Agents that make up this platform are in charge of tasks, like communication between heterogeneous systems, sending and receiving information (e.g., clinical reports, images, data sets, requirements, *etc*.), the management and storage of information and responding to requests in due time and in a correct way. It also provides tools to implement and to facilitate the communication with humans through web-based services [5,8]. AIDA was the first step for the so-called paper-free hospital, being the main repository of the electronic health record, also developed by researchers of the University of Minho. AIDA also implements important pre-processing procedures for data warehousing and clinical business intelligence.

**Figure 2:**
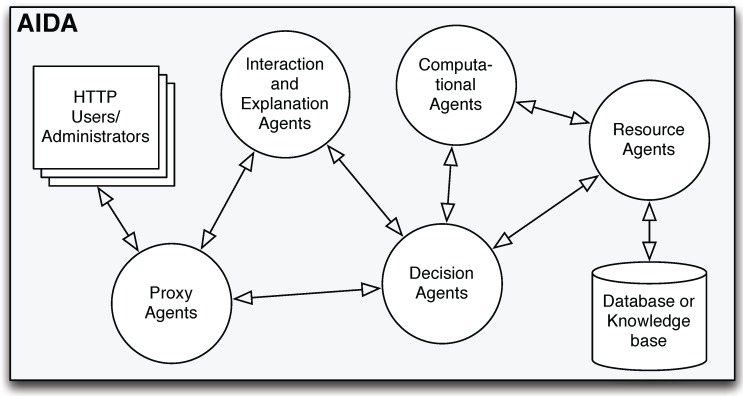
The Agency for Integration, Diffusion and Archive of Medical Information (AIDA) architecture adapted from [17].

Figure 2 presents the architecture model of the AIDA platform. As this figure demonstrates, there are five types of agents in AIDA [17]:

Proxy agents provide the connection between the system and the users/administrators. Through these agents, which communicate with the decision agents and the interaction and explanation agents, the users/administrators are able to:
  – access the information that they want to analyze;
  – obtain required explanations;
  – make decisions;
  – visualize the results in a web browser.
These operations are made through interfaces, monitoring dashboards and reports provided by AIDA to the users/administrators.
Decision agents act as mediators, accepting the tasks from the proxy agents. These agents have the ability to split these tasks into several sub-tasks, sending them to the computational agents for processing. After that, the decision agents receive the respective results from the computational agents. Decision agents also communicate with the interaction and explanation agents and the resources agents whenever it is necessary to process information based on argumentative proceedings or to access data stored in the databases, respectively.
Computational agents accept requests for specific tasks from the decision agents, returning the results after processing. These agents also communicate with the resources agents whenever specific information is required.
Resources agents have the ability to access specific data stored in the databases.
Interaction and explanation agents act based on argumentative proceedings that are fed with information coming from the proxy agents or the decision agents.

The EHR and all the IS (medical and nursing support IS, administrative, radiology, laboratory IS and others that belong to departments and services) are connected through the AIDA. Furthermore, the data center that processes and stores the information of the AIDA is connected to these systems, as well as the interfaces where users/administrators perform several operations. The agents shown in Figure 2 are responsible for archiving, managing and sharing the information related to the EHR and all the IS. In this way, each source of information communicates with another source of information through the messages exchanged among the AIDA agents, ensuring the interoperability of the platform [8]. 

After a brief study about AIDA’s advantages and disadvantages, it was possible to detect what weaknesses could be eliminated. The set of features shown below are the conclusions extracted from the study performed.

**AIDA Features**

• High power managing changes in the system;
• Object customization ability;
• High availability, accessibility and timely support;
• High security of the information and the system;
• Technologically modern;
• Ease of maintenance and simplicity of use of the system;
• Difficulty in the controlling agents and its activities.

Through these features, it can be concluded that the AIDA has many advantages. In spite of a few disadvantages, which have a weak impact in the AIDA workflow, this platform presents a high availability, accessibility and assistance in due time. Security is a very important issue, and AIDA ensures that the information about each individual is safe, and it respects certain ethical and legal standards. Overall, this platform is capable of providing all the information, to the authorized authorities, ensuring its integrity, confidentiality and availability [8].

The agents are the main core of AIDA. If a failure occurs in the execution of one of the agents, several consequences could happen. One of these can directly or indirectly affect the healthcare delivery to the patients. Therefore, the administrators that manage the AIDA platform should have total control of the agents. They should know when a specific agent is executing its tasks, how long it takes to accomplish its activity and other information. In this context, the necessity to create a module for controlling the agents community of AIDA arises. This module should present potentialities that allow the administrators to detect or prevent possible failures, to find their origin and to solve the problem.

## 5. Controlling the Agents Community of Archive of Medical Information (AIDA)

In order to overcome one of the drawbacks of the AIDA platform, the difficulty in controlling agents and their activities, which is common to most systems based on an MAS, the main features of a module for controlling the agents community of AIDA were studied. This module should allow the AIDA platform administrators to control and manage a community of agents, ensuring their survival in a heterogeneous environment.

According to the needs described by the administrators of the AIDA platform, several features of the module were stated, which aim to:

• Ensure greater control over the agents of AIDA;
• Facilitate the user’s work in the creation and registration of new agents, locally or remotely;
• Allow the user to enable and to disable services at the health unit, through the launch or stop of a particular agent;
• Facilitate the scheduling and rescheduling of the agent’s activity;
• Monitor dynamically and in real time the activity of the agents;
• Send alerts or warnings by email;
• Perform auditing procedures.

The auditing procedures and the trigger alert by email are ensured by a monitoring and preventing system developed in parallel [37] and subsequently integrated with the module. This system is based on a forecasting model used in medicine: Modified Early Warning Score (MEWS) [38,39]. This model is used to predict serious health problems, assuming that these problems are often preceded by physiological deterioration. The MEWS implies a strict monitoring of the patient’s vital signs [37,38]. Through a decision table, scores are attributed to seven parameters, such as: temperature, heart rate, systolic blood pressure, respiratory rate, blood oxygen, urine output and neurological symptoms. In this way, it is possible to determine the level of risk of each patient, preventing organ failures, understanding the behavior of the vital signs over time and assisting in making medical decisions [38,39].

The monitoring and preventing system developed in parallel with the work presented in this paper [37] is based on MEWS. However, in this case, instead of patients, the agents and the machines of AIDA are monitored and risk situations are detected, preventing damage to the AIDA workflow. It verifies periodically the agents’ performance (CPU, RAM and frequency with which the agents execute their activities). In this way, this system detects situations conducive to failure, and it warns the administrators (via email) to take preventive actions, avoiding damage to the AIDA workflow. This system also monitors the performance and prevents failures in the AIDA machines [37].

This module should be based on a client-server architecture, where the server is able to communicate with multiple clients simultaneously through the use of one thread dedicated to each client. Each new agent corresponds to a new client, and the ACL messages are exchanged among the agents through sockets. The sockets make the use of the TCP/IP protocol to ensure that information is transferred from an application/agent to another, keeping the integrity of the transferred data.

**Figure 3:**
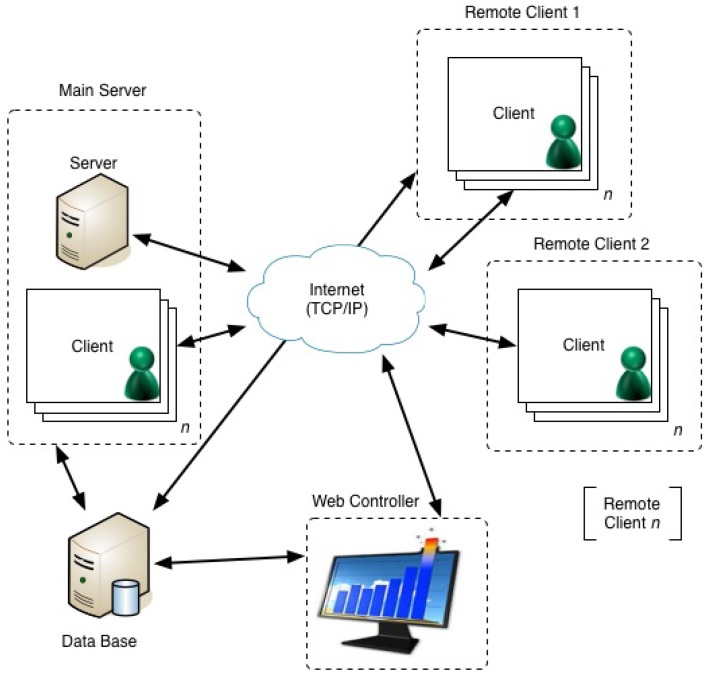
Architecture of the module for controlling the agent community of the Agency for Integration, Diffusion and Archive of Medical Information (AIDA).

Analyzing the module architecture presented in Figure 3, it is possible to verify that it is composed of three distinct components: the main server, the remote clients and the web controller. This module is defined as a tuple, Ξ *≡ 〈*∆*_module_*, *MainServer*, *WebController*, *RemoteClient*_1_, ..., *RemoteClient_n_〉*, where:

∆_*module*_ is a set of bridge rules that establish the communication/interaction among all components of the module;
*MainServer* is composed of *〈server*, *a_AMS_*, *a*_1_, ..., *a_n_〉*, where *server* is the server for the entire system, *a_AM S_* is the agent that manages all agents hosted on the machine where the main server is located and *a*_1_, ..., *a_n_* are the AIDA agents that perform their activities in the same machine;
*WebController* is a user interface to control the agents, which allows the user to schedule the activities of each agent and also to monitor their activities;
*RemoteClient*_1_, ..., *RemoteClient_n_* are composed of *〈a_AM S_*, a_1_, ..., *a_n_〉*, and their purpose is to connect all the machines that constitute the module.

Security and privacy are guaranteed by a set of protocols that control the access and maintain information privacy. These protocols are implemented in the whole AIDA platform, including this new module, and these do not allow unauthorized users to access information that is manipulated by the agents. In addition, the CHP has implemented security protocols among all TCP/IP connections, including the ones established in the AIDA platform. It is also important to mention that all the information entered into the AIDA databases is ensured by mechanisms that endure failure situations, such as the Real Application Clusters provided by Oracle and a data guard solution [40]. In this way, all the information generated in the AIDA platform is confidential, available and protected [8].

### 5.1. Main Server and Remote Clients

**Figure 4:**
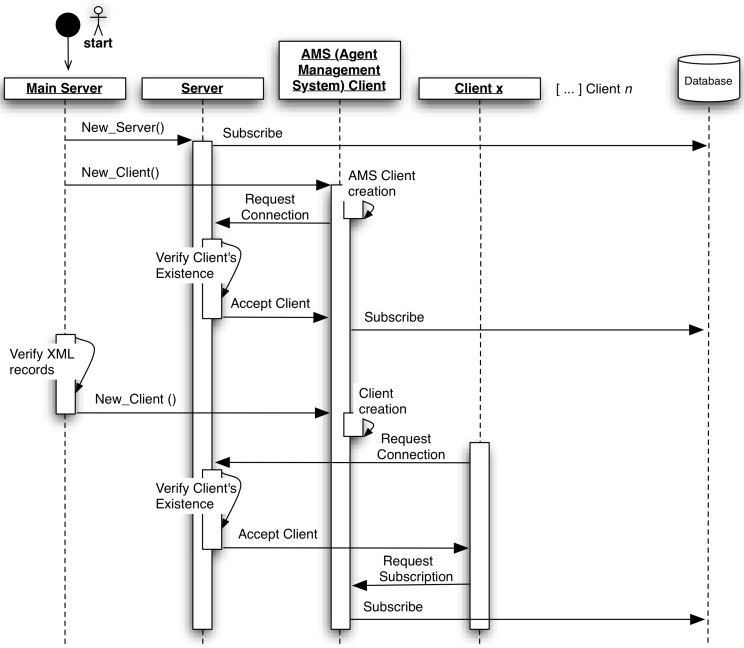
Automatic boot process of the module for controlling the agent community of Agency for Integration, Diffusion and Archive of Medical Information (AIDA).

The main server component is used to initiate the module (Figure 4) automatically, and once it is the server, it can only be executed on the main machine. On the other hand, the remote clients are executed in all other machines that host agents.

Firstly, a server is created in a specific IP and port, and then, it subscribes itself to a database, which store all information about this module. In this way, future clients will have the possibility of accessing the database and extracting the server IP and port in order to connect to it.

All machines need to have an Agent Management System (AMS) agent that is responsible for controlling the other agents that are in the same machine. Analyzing Figure 4 minutely, the first agent/client that is created is the AMS. Besides that, after its creation, the AMS connects to the server after a proper request and verification. To make the AMS operable, it subscribes itself to the database, recording its name, its state (active or inactive) and the machine IP wherein it operates.

In each machine, the module developed creates an XML file in order to save information about the hosted agents. The purpose of this file is to store information locally about the agents that are active. In this way, when it is necessary to realize the boot process (for example, when the machine or the module is restarted), the administrator does not need to start the agents manually, like the first time, when the module was initiated. Instead, the agents are automatically started after a proper verification of the XML file content. Continuing the analysis of the Figure 4, the only difference between the AMS creation and the other agents/clients creation is that the agents/clients are not able to subscribe themselves to a database; they need to send a request to their own AMS.

Despite this, the module can work in a single machine just with the main server; it is important to note that this component is also able to control agents in other machines through the remote clients, keeping the communication among all agents regardless of the machine in which they are executed. The remote clients are started like the main server, with the exception of the server creation. In these cases, the request connection is sent to the server situated in the main machine, where the main server component is.

### 5.2. Web Controller

The web controller aims at providing an attractive environment to control and to monitor the agents subscribed to the module. This component is a web interface that can be accessed ubiquitously through any computer.

This component has a main page that enables the user to analyze the module overview; specifically, the agents that are subscribed and their physical localization. Exploiting each agent, it is possible: to visualize its properties; to schedule its activities; and to monitor its performed activities in real time and in a dynamic way.

The web controller property page enables the user to consult the list of all properties of each agent. In addition to the properties, it is possible to know the details about the scheduling of the agent: how and when it was scheduled. On this page, an overview of the activities performed by the agent from its creation until the present moment is available.

The scheduling page enables the user to schedule, reschedule and cancel the agent activity. The scheduling can be done for the agent to execute its activity:

• At a determined hour of the one or more days selected in a calendar by the user;
• Daily, at a determined hour;
• Weekly, at a determined hour, on a specific day of the week;
• Monthly, at a determined hour, on a specific day of the month;
• In a specific interval (seconds, minutes, hours or days).

Finally, the monitoring page offers the user a set of two kinds of graphs that enables the control of the activity of all agents. For each kind of graph are shown three analyses: a daily, a weekly and a monthly. The graph of the duration of the agent activity shows the average time that the agent takes to perform its activity in seconds. The daily monitoring shows this average by the hour of the day selected in the calendar, weekly by the day of the week in which the selected day is inserted and monthly by the day of the month of the selected day. Similarly, the graph of the number of executions of the agent presents these three analysis, and it shows the number of times that the agent performs its activity, by hour, day of the week and day of the month.

Once the web controller is a web component, it can be accessed through any device that has a browser anywhere and anytime. Besides that, all the information used to construct these web pages is stored in the AIDA, which ensures the information’s security (confidentiality, integrity and availability).

## 6. Results

The module for controlling the AIDA agent community was implemented in a real environment, in the Centro Hospitalar do Porto (CHP), in order to evaluate its performance. A set of three agents was created in this new module, and their activities were monitored during the period between 10 and 16 September 2013. Due one of the agents have more of a workload, the results of Agent 609 were selected to be shown in this paper. This agent, responsible for ensuring the interoperability with the information system in which are registered the data resulting from nursing practices, was scheduled to execute its activity every 10 min.

**Figure 5:**
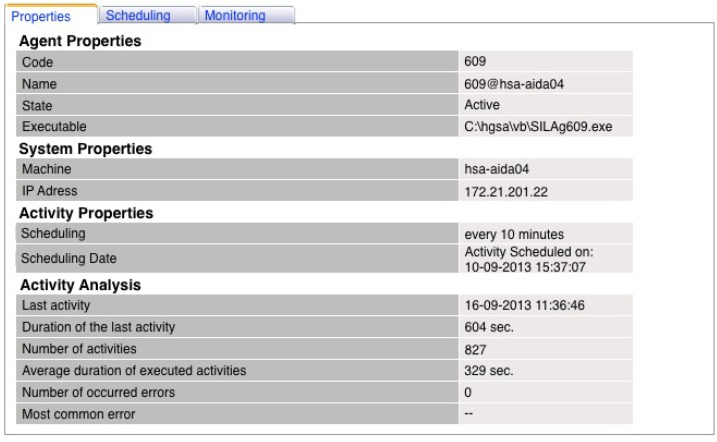
Properties page from Agent 609.

Figure 5 represents the properties page from Agent 609. This page enables the user to know the agent code and its name; the last one is composed of the code and the machine name where the agent performs its activities. Its state is also shown and can be “active” when the agent is registered on the module and “inactive” when it was registered, but currently is not registered. The executable field corresponds to the path of the logic of the agent that runs according to the schedule made by the user. There is one executable per agent, and it is stored locally on the machine where the agent is hosted. It is possible to see that the agent was scheduled on 10 September 2013, at 3:37 p.m. Since, then it was executed 827 times, with an average of 329 s without errors. The last execution of Agent 609 had been on 16 September 2013, at 11:36 a.m., and it had a duration of 604 s.

It is important to mention that the average duration was calculated using the 50th percentile in order to present a measure of central tendency, thus eliminating some outlines. With this information, the user can also reach other interesting findings; for example, one may also calculate the number of times the agent has performed its activity for a day, *i.e.*, if the agent has performed its activity 827 times for nearly six days, per day performed, approximately 137 times, and by hour, six times.

**Figure 6:**
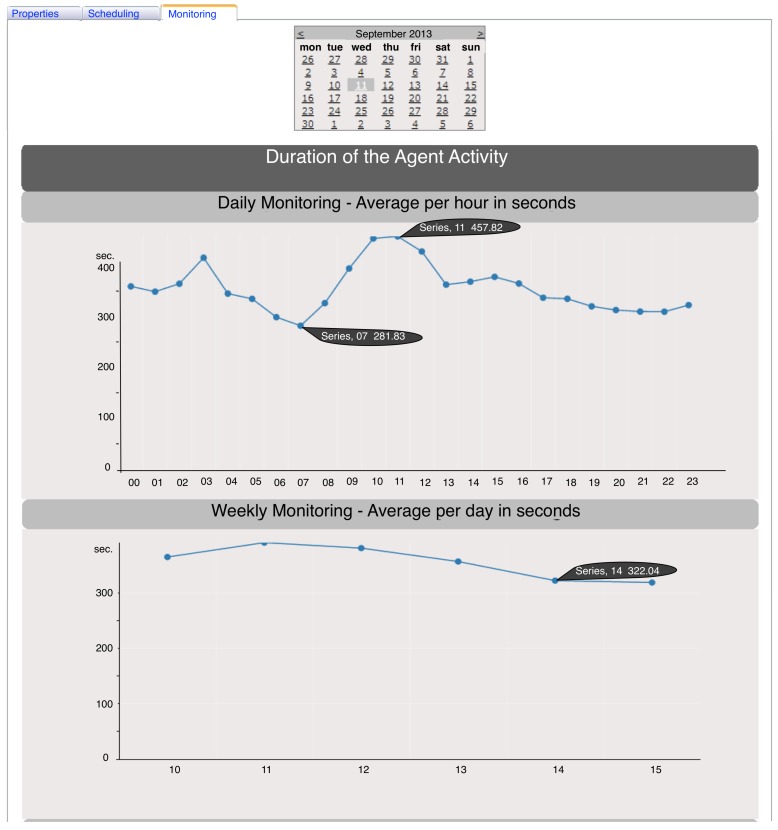
Daily and weekly analysis of the duration of an agent activity on 11 September 2013.

Apart from the data that the properties page provides (described above), the web controller generates graphics, such as that shown in Figure 6. After selecting the day that the user wants to analyze, these graphs are built (the third graph in this case is not shown, because the analysis period corresponds to only one week). Analyzing in detail Figure 6 (daily monitoring), it is concluded that the agent took less time to execute its tasks between 7 a.m. and 8 a.m., with an average of 281.83 s. The maximum value was detected between 11 a.m. and 12 p.m., with a duration average of 457.62 s. It is possible to verify an abrupt growth between 8 a.m. and 12 p.m. followed by a gradual decrease until 11 PM, which could be related to the volume of data registered on the nursing information system and, consequently, with the influx of patients at the healthcare institution. It can also be checked that there exists a peak in the dawn, at 3 a.m., that may be due to an emergency situation or a maintenance action that, for the CHP, are generally performed during this period.

Similarly, in the weekly monitoring, still in Figure 6, the user can observe the weekly flow and find out which are the days that the agent takes to carry out its activities. For the week in which 11 September 2013 is inserted, it can be noted that on the weekend, the agent performs its activities much faster (322 s) than the other days of the week (about 350 s), and Wednesday is the day with the highest mean value (390 s).

With this new module integrated with the AIDA and implemented in the CHP, the AIDA platform administrators may better manage the whole platform through the monitoring of the agents that constitute it. Therefore, this module provides all the needed information to know what period and what days the agents have more functions, *i.e.*, that their activity is more time consuming. Thus, it is possible to schedule routine changes, updates and other operations, improving the overall performance of the platform without disrupting the proper functioning of the agents and, consequently, of the health institution.

Through these graphs and all the functionalities provided by the web controller, it is possible to guarantee interoperability in the AIDA platform. For instance, the monitoring and controlling of Agent 609 ensures the correct communication between the AIDA platform and the system responsible for nursing practices, ensuring a proper interoperability procedure.

**Figure 7:**
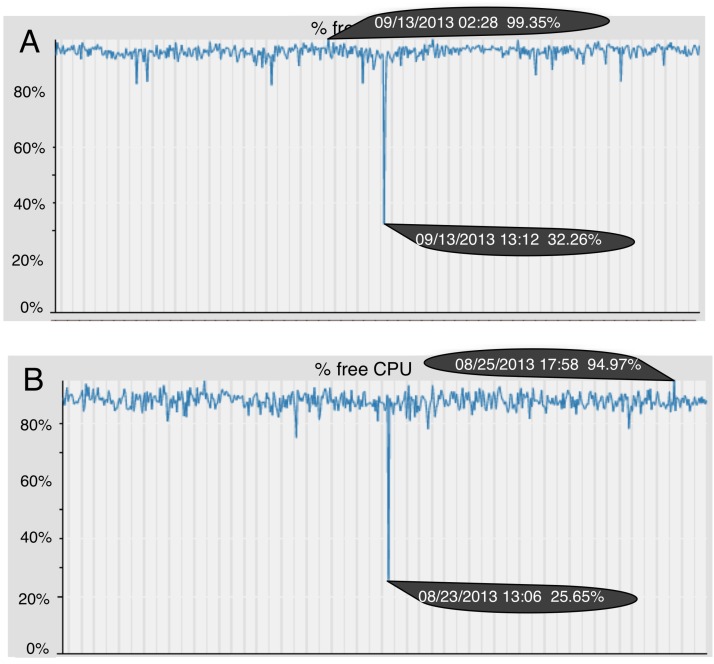
Comparison of the percentage of free CPU: (**A**) the period of the module implementation (11 to 15 September 2013); (**B**) the period preceding the implementation (21 to 25 August 2013).

To check the efficiency of this new module, *i.e.*, to ensure that this module does not compromise the machine resources, a monitoring and fault forecasting system was used [37] to monitor the machine resources (CPU and RAM). Among other things, this system provides graphs with the percentage of free CPU and RAM, as presented in Figure 7. Using this system in the period of the implementation of the new module (Figure 7A) and in a previous period (Figure 7B), it is possible compare the percentage of free CPU.

Comparing the two graphs, it is possible to verify that there was a slight increase in the percentage of free CPU in the implementation period. It can be seen that in Figure 7A, the highest peak of the free CPU is 99.35%, whereas in Figure 7B, it is 94.97%. The abrupt depression occurred on the same day of the week at the same time as the peaks, but the value in Figure 7A is greater than in Figure 7B. With the percentage of free RAM, the scenario was identical. This allows one to conclude that the resources of the machines are not committed when this new module is used.

This set of results reveals that the module for controlling the agent community of AIDA offers to AIDA a greater functionality, once it promotes functions that satisfy the users’ needs. Besides that, the module also offers more usability to AIDA, since it is an easy to understand and operate system with an attractive interface.

**Table 1:** Comparison of several solutions. FIPA-ACL, Foundation for Intelligent Physical

Solution	Field	Communication in the MAS	Agents migration	Agents monitoring	Agents activities scheduling	Observations
Marques [23]	e-Government	Messages structure notspecified.	X	X	X	Prototype of an interoperability architecture.
Contreras [25]	Any field, tested in the oil industry.	FIPA-ACL; XML for encoding.	The agents can be static ormobile.	It monitors the state, properties and logs of the agents.	X	X
Lanzola [28]	Healthcare	ACL	X	Solving the control problems using meta-rules.	X	Prototype of aframework for cooperative agents
Orgun [30]	Healthcare	HL7 messages	Mobil eagents	X	X	X
Kim [32]	u-Healthcare	Messages structure notspecified.	Mobile agents	Logs monitoring	X	X
New AIDA module	Any field, tested in the healthcare.	FIPA-ACL	X	It monitors the state and properties of the agents. It stores and enables one to analyze the performance of theagent activities.	It enables agent scheduling and rescheduling if necessary.	X

Comparing the solution presented in this paper with others presented in Section 3 (Table 1), it is possible to state that our solution is endowed with great scalability and adaptability, once new agents can easily be added to the system, and it can be applied to any field (unlike most of the solutions presented). In spite of our solution not supporting mobile agents, it provides proper management and customization of the agents through an intuitive and dynamic interface. This interface owns scheduling functionalities and monitoring dashboards, which aid the system administrators to manage and to ensure interoperability in the AIDA platform.

This new module was integrated with AIDA in order to ensure an efficient communication among the agents, being considered a good practice for interoperation and communication in an MAS. The scientific community that uses intelligent agents to solve a wide range of problems in the informatics area should look into this solution as a base for the construction of an MAS, once the MAS included in this new module of AIDA was revealed to be very important for the AIDA platform implemented in the CHP. The importance of this module in the CHP was evidenced, allowing the full control of AIDA agents. This full control enables the AIDA administrators to perform better management of the platform, not overloading the workflow of AIDA and of the HIS and, therefore, ensuring the quality of the services delivered.

## 7. Conclusions

This project is part of an important theme these days: interoperability. Over the past few years, it has emerged as a requirement in most health services entities. The AIDA platform has been revealed as a huge success regarding the interoperability among HIS through its main functions, the integration, the archiving and the diffusion of medical information, which are ensured by its multi-agent system [17].

Using the agent-oriented programming, the ACL messages and an architecture client/server multi-thread with TCP/IP sockets, it was possible to develop the module for controlling the agent community of AIDA components.

The results presented allow the users to analyze in a detailed way the activity of each agent. Through the web controller, dynamic graphs with the average duration and the number of executions of the agents are available.

Nevertheless, the main scientific contribution of this module that controls an agent community is the fact that it is transferable and operable in other application domains (e.g., banking, insurance, municipal services and many others). In other words, it is a system that can be applied to other multi-agent systems without wasting much time in resetting the module. Through this module, the user/administrator of the multi-agent system can create, subscribe and schedule their agents in a simple way. Furthermore, the activation and deactivation of services through the launch and stop of agents is facilitated, and the module also allows the monitoring of all activities dynamically and in real time. It is important to note that the Modified Early Warning Score (MEWS) approach used in the work developed together with this module [37] is revealed to be a great scientific contribution regarding the procedures for monitoring and preventing in the HIS.

It is possible to conclude that the module for controlling the agent community of AIDA implemented in CHP offers to AIDA a greater functionality and usability, ensuring interoperability in a reliable way with great scalability and growth capacity. This module, although a specific module of a platform, can be adapted and extended to other platforms in healthcare that use agents. The same can be used as a top layer of the management and evaluation of agents. This module represents an important contribution to the next generation of interoperability in healthcare, ensuring continuous data collection from heterogeneous data sources in order to support the decision-making process in real time. This work is essential for the proper functioning of new platforms in healthcare.

As future work, new graphs will be built in order to give to the user more information about the agent activities. The communication between the module for controlling the agent community of AIDA and a monitoring and fault forecasting system developed by our research group [37] is being planned. The purpose of this communication is to enable the balancing of resources automatically through agent migration. To implement this procedure, the creation of a repository will be required to store the logic of the agents.
